# Practical Machine-Checked Formalization of Change Impact Analysis

**DOI:** 10.1007/978-3-030-45237-7_9

**Published:** 2020-03-13

**Authors:** Karl Palmskog, Ahmet Celik, Milos Gligoric

**Affiliations:** 8 Johannes Kepler University, Linz, Austria; 9 University of Birmingham, Birmingham, UK; 10 KTH Royal Institute of Technology, Stockholm, Sweden; 11 Facebook, Seattle, WA USA; 12 The University of Texas at Austin, Austin, TX USA

**Keywords:** Change impact analysis, Regression test selection, Coq

## Abstract

Change impact analysis techniques determine the components affected by a change to a software system, and are used as part of many program analysis techniques and tools, e.g., in regression test selection, build systems, and compilers. The correctness of such analyses usually depends both on domain-specific properties and change impact analysis, and is rarely established formally, which is detrimental to trustworthiness. We present a formalization of change impact analysis with machine-checked proofs of correctness in the Coq proof assistant. Our formal model factors out domain-specific concerns and captures system components and their interrelations in terms of dependency graphs. Using compositionality, we also capture hierarchical impact analysis formally for the first time, which, e.g., can capture when impacted files are used to locate impacted tests inside those files. We refined our verified impact analysis for performance, extracted it to efficient executable OCaml code, and integrated it with a regression test selection tool, one regression proof selection tool, and one build system, replacing their existing impact analyses. We then evaluated the resulting toolchains on several open source projects, and our results show that the toolchains run with only small differences compared to the original running time. We believe our formalization can provide a basis for formally proving domain-specific techniques using change impact analysis correct, and our verified code can be integrated with additional tools to increase their reliability.

### 1 Introduction

Change impact analysis aims to determine the components affected by a change to a software system, e.g., the modules or files affected by a modified line of code [3,4]. Change impact analysis techniques are used in many program analyses and tools, such as regression test selection (RTS) tools [26, 52, 59, 61], build systems [15, 21, 43, 45], and incremental compilers [48].

Change impact analysis techniques typically mix domain- and language-specific concepts, such as method call graphs and class files, with more abstract notions, such as dependencies, transitive closures, and topological sorts. This can complicate reasoning about the correctness (*safety*) of a technique. For example, to the best of our knowledge, RTS techniques for Java-like languages have never been argued to be safe (i.e., to never omit tests affected by a change) by *machine-checked reasoning*—only by high-level pen-and-paper proofs [51,55,60].

In this paper, we present a formalization of key concepts used in many change impact analysis techniques—concepts that are *independent of any language* or application domain. Our formalization represents system components and their interrelations as vertices and edges in explicit *dependency graphs*. We consider whether components are *impacted* by changes between two system revisions by computing transitive closures of modified graph vertices in the *inverse* of the dependency graph from the old revision. This has been described as "invalidating the upward transitive closure" [14]. Among impacted vertices, we identify those that are *checkable*, representing, e.g., a test method, that can be re-executed.

We encoded our formal model as a library in the Coq proof assistant, and proved two key correctness properties: *soundness* and *completeness*. Soundness, intuitively, states that the outcomes of executing checkable vertices that are unimpacted in the new revision are the same as they would be in the previous revision. Completeness roughly states that all checkable vertices in the new revision are members of the set of all added, impacted, and unimpacted vertices.

Based on our correctness approach, we also defined and proved correct two strategies for *hierarchical* change impact analysis that are roughly analogous to, on the one hand, file-based incremental builds [43, 54], and on the other hand, hybrid regression test selection [46, 60]. To the best of our knowledge, hierarchical change impact analysis is previously unexplored in formal settings like ours. Ultimately, by proving some basic properties about relations between vertices and results of executing checkable vertices, developers can use our model and library to obtain end-to-end guarantees for domain-specific impact analyses.

To capture our model of system components and their dependencies in Coq, we used the Mathematical Components (MC) library [42] and its representation of relations, finite graphs, and subtypes [25,28,29]. For the formal proofs, we used the SSReflect proof language and followed the idiom of the MC library of leveraging boolean decision procedures in proofs via *small-scale reflection* [9, 30, 31]. To obtain efficient executable code, we performed several verified refinements of our initial Coq encoding. From our refined functions and datatypes, we then derived a practical tool, dubbed Chip, by carefully extracting Coq code to OCaml and linking it with an assortment of OCaml libraries. Chip can be viewed as a *verified component* for change impact analysis that can either be integrated into verified systems or used in conventionally developed systems.

To ensure the adequacy of our formal model, we performed an empirical study using Chip. Specifically, we integrated Chip with Ekstazi [26], a tool for class-based regression test selection in Java, with iCoq [11], a tool for regression proof selection in Coq itself, and with Tup [54], a build system similar to `make`, replacing the existing components for change impact analysis in all these tools. We then compared the outcome and running time between the respective modified and original tool versions when applied to the revision histories of several open-source projects. This approach is along the lines of previous evaluations of formal specifications [8, 20, 33] and RTS techniques [26, 37, 60]. During our evaluation of Chip, we also located and addressed several performance bottlenecks.

We make the following contributions:

– **Basic formal model**: We present a formalization of change impact analysis in terms of finite graphs and sets, encoded in the Coq proof assistant via the MC library. We formulated and proved in Coq key correctness requirements for our analysis, namely, soundness and completeness.
– **Hierarchical formal model**: We extended our model to capture two strategies for hierarchical change impact analysis, where higher-level components are implicitly tied to lower-level components, and proved them both correct.
– **Library**: Our Coq development forms a library of definitions and lemmas that can assist in formally proving various techniques based on change impact analysis, such as regression test selection for Java, correct inside Coq.
– **Optimizations**: We refined our verified Coq functions and data structures to significantly improve performance in practice of code extracted to OCaml.
– **Tool**: From our refined Coq code, we derived a verified executable tool in OCaml for change impact analysis, Chip, by carefully leveraging Coq's code extraction mechanism. Chip can be used as a *verified component* for both regular and hierarchical change impact analysis in other tools. The Chip code, compatible with Coq 8.9, MC 1.7, and OCaml 4.07, is publicly available [47].
– **Evaluation**: We integrated Chip with a tool for regression test selection in Java projects, Ekstazi, one regression proof selection tool for Coq itself, iCoq, as well as one build system, Tup, and evaluated the resulting toolchains on several medium to large-scale open source projects.

### 2 Background

In this section, we give some brief background on change impact analysis and its applications, and on the Coq proof assistant.

#### 2.1 Change Impact Analysis

Broadly, we consider change impact analysis as the activity of identifying the potential consequences of a change to a software system. Formulated in this way, change impact analysis is an old concern in software engineering [4], and remains an active research topic as part of techniques and tools [1, 34, 53]. In early work, Arnold posited computing transitive closures of statically derived program call graphs as the fundamental technique for change impact analysis [3]. However, later research argues that dynamic analysis can be more precise [36] and lead to faster dependency collection for use in future analyses [26]. Our work aims to capture general concepts used in both static and dynamic approaches [10, 38].

#### 2.2 Regression Test Selection and Regression Proof Selection

Regression test selection (RTS) techniques optimize regression testing – running tests at each project revision to check correctness of recent changes – by deselecting tests that are not affected by the recent changes [50, 59]. Traditionally,

RTS techniques maintain for each test a set of code elements (e.g., statements, methods, classes) on which the test depends. When code elements are modified, change impact analysis is used to detect those tests that are potentially affected by the changes. Prior work has studied RTS for various programming languages (e.g., C, C++, and Java), built dependency graphs statically or dynamically, and used various granularities of code elements (e.g., statements, methods, and classes). The meaning of the dependency graph is language-specific, but if the graph is properly constructed, the change impact analysis is independent of the language. For example, Ekstazi [26], a recent RTS tool for Java projects, builds and maintains Java class file dependency graphs dynamically, and when a class file is modified, Ekstazi uses change impact analysis to select all test classes that depend, directly or indirectly, on the modified class.

Regression proof selection (RPS) is the analogue of RTS for formal proofs, which, similarly to tests, can take a long time to check. The RPS technique implemented in the iCoq tool for Coq [12] uses *hierarchical* selection [11], where impacted files are used to locate impacted proofs to be checked.

#### 2.3 Build Systems

The classic build system `make` uses file timestamp comparisons to decide whether a task defined in a *build script* should be run. Dependency graphs are implicitly defined by tasks depending on the completion of other tasks, or on certain files, as expressed in the build script. In contrast to test execution, build script task execution typically produces side effects in the form of new files, e.g., files with object code in ELF format. Modern build systems such as Bazel [5] and CloudMake [21,27] can use other ways than timestamps to find modified files, e.g., comparing cryptographic hashes of files across revisions. Recent alternative build systems that aim to replace `make` include Tup [54] and Shake [43]; the former uses an explicit persisted dependency graph.

#### 2.4 The Coq Proof Assistant and Mathematical Components

Coq consists of, on the one hand, a small and powerful purely functional programming language, and on the other hand, a system for specifying properties about programs and proving them [6]. Coq is based on a constructive type theory [17,18] which effectively reduces proof checking to type checking, and puts programming on the same foot as proving. Mathematical Components (MC) [42] is an extensive Coq library that provides many structures from mathematics, including finite sets, relations, and subtypes; we use the module `fingraph`, which was derived from Gonthier's proof of the four-color theorem [28].

Datatypes and functions verified inside Coq to have some correctness property can be *extracted* to a practical programming language such as OCaml [40], and then integrated with libraries; extraction is used in several large-scale software verification projects [39,57]. Obtaining efficient programs via extraction may require significant engineering because of discord between the requirements for formal correctness and agreeable program runtime behavior [19]. When target languages lack fully formal semantics, as is the case for OCaml, extraction cannot be fully trusted, but empirical evaluations are nevertheless encouraging [24,58].

### 3 Formal Model

This section introduces our model, assumptions, and correctness approach.

#### 3.1 Definitions

**Components**: Our model of change impact analysis uses two finite sets of vertices $V$ and $V'$, where $V \subseteq V'$. Members of these sets represent the components of a system (e.g., files or classes) before and after a change, respectively.
**Artifacts**: We let $A$ be a set of *artifacts*. An artifact is intended to be a concrete underlying representation of a component, e.g., an abstract syntax tree or the content of a file. We assume that the equality of two artifacts is decidable, i.e., that we can compute for all $a, a' \in A$ whether $a = a'$ or $a \neq a'$. To associate vertices with artifacts, we use two total functions $f \colon V \to A$ and $f' \colon V' \to A$. In practice, we expect these functions to map vertices to compact *summaries* of component representations, such as checksums computed by cryptographic hash functions. Whenever $f(v) \neq f'(v)$ for some $v \in V$, we say that the artifact for $v$ is *modified* after the revision; otherwise, it is *unmodified*.
**Graphs**: Let $g$ be a binary relation on $V$. For $v, v' \in V$, we say that $v$ *directly depends on* $v'$ if $g(v, v')$ holds. For example, if $v$ and $v'$ represent classes in a Java-like language, $v$ may be a subclass of $v'$. We will usually refer to relations like $g$ as (dependency) *graphs*. We write $g^{-1}$ for the inverse of $g$, i.e., we have $g^{-1}(v, v')$ iff $g(v', v)$. Moreover, we write $g^*(v, v')$ for when $v$ and $v'$ are transitively related in $g$, and say that $v$ *transitively depends on* $v'$. We define the *reflexive-transitive closure* of a vertex $v \in V$ with respect to a graph $g$ as the set $\{v' \mid g^*(v, v')\}$, i.e., as the set of all vertices reachable from $v$ in $g$ (which includes $v$ itself).
**Execution**: We assume there is a subset $E \subseteq V'$ of *checkable* vertices, i.e., it is meaningful to apply some (side-effect free) function *check* on them and obtain some result. For example, a checkable vertex may represent a test method that either passes or fails when executed.
**Impactedness**: Let $g$ be a dependency graph. We then say that a vertex $v \in V$ is *impacted* if it is reachable in $g^{-1}$ from some modified vertex. Equivalently, $v$ is impacted iff there is a $v' \in V$ such that $f(v') \neq f'(v')$ and $(g^{-1})^*(v', v)$. Additionally, a vertex $v'' \in V'$ is considered *fresh* whenever $v'' \notin V$.

We take the (disjoint) union of the set $I$ of impacted vertices and the set $F$ of fresh vertices, and consider the checkable vertices in this set, i.e., vertices in $\big(I \cup F\big) \cap E$. Intuitively, these are the only vertices that we need to consider in the new revision, since all other vertices in $V'$ are *unimpacted*—and using *check* on unimpacted vertices will have the same outcome as in the old revision.

#### 3.2 Example

Figure 1 illustrates the core idea of the graph-based change impact analysis approach we model. Figure 1(a) shows the original dependency graph, where, e.g., component 3 depends directly on components 1 and 2, and 5 depends directly on 3 and transitively on 1 and 2; dotted components are checkable. Figure 1(b) shows the inverse graph, with the modified component 1 bolded, and the components impacted by the change in gray (the reflexive-transitive closure of 1 in the inverse graph). Based on these results, we call *check* on 5, but not on 6.

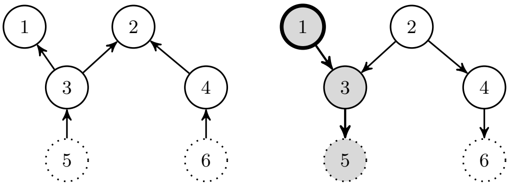

**Fig. 1.** Dependency graph where component 1 is changed, impacting 3 and 5.

#### 3.3 Correctness Approach

For correctness, we intuitively show that executing only impacted and fresh vertices that are checkable is enough in the new revision, since the result of executing unimpacted vertices is the same as in the old revision. This means that if we have access to the results of checking vertices in the old revision, we can use those results to obtain the complete outcome for all checkable vertices in the new revision, without going through the work usually required.

Having constructed the set $T$ of tuples of checkable vertices and outcomes from the impacted, fresh, and unimpacted vertices, we can ask (1) whether $T$ is *complete*, i.e., whether it contains outcomes for all checkable vertices in $V'$, and (2) whether the outcomes in $T$ are *sound*, i.e., if they are same as if we had explicitly called *check* on the associated vertices.

To be able to prove soundness and completeness, we need to assume several properties relating the dependency graphs and outcomes of executing vertices in both revisions. Informally, we make the following assumptions:

A1: The direct dependencies of a vertex $v$ are the same in both revisions if the artifact of $v$ is the same in both revisions, i.e., if $f(v) = f'(v)$.

A2: A vertex $v$ with the same artifact in both revisions is checkable in the new revision iff $v$ is checkable in the old revision.

A3: The outcome of executing a checkable vertex $v$ is the same in both revisions if the sets of vertices $v$ depends on transitively are the same, and the artifact of each dependency is the same.

The last assumption implicitly rules out that the underlying operation (e.g., test execution) on a vertex is *nondeterministic*, which it can be in practice [41].

### 4 Model Encoding

In this section, we give an overview of our encoding in Coq of the formal model described in the previous section, using theories of finite sets and graphs from the MC library. We use a simplified version of Coq's specification language, Gallina.

#### 4.1 Encoding in Coq

We represent the vertex set $V'$ as a *finite type* (`finType`) `V'`, and its subset $V$ as a *subtype* (`subType`) `V`, induced by a decidable predicate `P` on vertices in `V'` (of type `pred V'`). This allows us to define the graph $g$ as a binary decidable relation `g` on `V`, i.e., a variable of type `rel V`, and use the MC library predicate `connect`

to express whether two vertices are transitively related in `g`. The inverse of `g` is defined as `[rel x y | g y x]`, which we write as $g^{-1}$. We use `connect` to form the set of vertices in the reflexive-transitive closure of a given vertex `x` with respect to a graph `g`, and a canonical big operator [7] to form the union of all such closures for elements in a given set `m` of modified vertices:

```
Def impacted (g : rel V) (m : {set V}) : {set V} :=
 \bigcup_( x | x \in m) [set y | connect g x y].
```

We characterize this function through MC's `reflect` ("if and only if"):

```
Thm impactedP g m x : reflect (∃ v, v \in m & connect g v x) (x \in impacted g m).
```

The MC library function `val` injects a subtype element into the corresponding supertype. We use this to capture impacted and fresh vertices in `V'`:

```
Def impacted_V' m : {set V'} := [set (val v) | v in impacted g⁻¹ m].
Def fresh_V' : {set V'} := [set v | ¬ P v].
```

We represent the set of artifacts $A$ as a type `A` with decidable equality (`eqType`), and functions $f$ and $f'$ as regular Coq functions `f` and `f'`. This allows us to define the set of modified vertices in `V'`, and then take the union (operator :|:) of impacted and fresh vertices:

```
Def mod_V : {set V} := [set v | f v != f' (val v)].
Def impacted_fresh_V' : {set V'} := impacted_V' mod_V :|: fresh_V'.
```

We then use a predicate `checkable` to form the subset of vertices in `V'` that can be executed:

```
Def chk_impacted_fresh_V':{set V'} := [set v in impacted_fresh_V' | checkable v].
```

We use a function `check`, which takes a vertex and returns a term in a result type `R` (an `eqType`, e.g., `bool`), to define a sequence of vertices and results:

```
Def res_impacted_fresh_V' : seq (V' * R) :=
 [seq (v, check v) | v ← enum chk_impacted_fresh_V'].
```

Note that by using a sequence instead of a finite set for these tuples, we ensure `R` can be any type with decidable equality, such as a message of arbitrary length.

### 4.2 Correctness Statements

For stating and proving correctness, we assume we have dependency graphs for the old and new revision, as well as definitions of whether vertices are checkable, and checking functions:

```
Vars (g : rel V) (g' : rel V').
Vars (checkable : pred V) (checkable' : pred V') (check : V → R) (check' : V' → R).
```

We then define the graph `g` for vertices in `V'`, named `g_V'`:

```
Def insub_g (x y : V') : bool := match insub x, insub y with
 Some x', Some y' ⇒ g x' y' | _, _ ⇒ false end.
Def g_V' : rel V' := [rel x y | insub_g x y].
```

This allows us to formulate the assumption A1 from above:

```
Hyp fg_eq : ∀ (v : V), f v = f' (val v) → ∀ (v' : V'), g_V' (val v) v' = g' (val v) v'.
```

The assumption A2 is equally straightforward to define:

```
Hyp chk_f : ∀ v, f v = f' (val v) → checkable v = checkable' (val v).
```

Finally, the assumption A3, when formalized, establishes a relation between vertices in `g` and `g'`:

```
Hyp chk_V : ∀ (v : V), checkable v → checkable' (val v) →
 (∀ (v' : V'), connect g_V' (val v) v' = connect g' (val v) v') →
 (∀ (v' : V'), connect g_V' (val v) (val v') →
  f v' = f' (val v')) → check v = check' (val v).
```

We now assume we are given a sequence of results for checkable vertices in the old revision, and that this sequence is sound, complete, and duplicate-free:

```
Var res_V : seq (V * R).
Hyp res_VP : ∀ v r, reflect (checkable v ∧ check v = r) ((v,r) \in res_V).
Hyp res_v_uniq : uniq [seq vr.1 | vr ← res_V].
```

We can then filter the sequence of old results to locate unimpacted vertices in the new revision:

```
Def res_unimpacted_V' : seq (V' * R) := [seq (val vr.1, vr.2) |
 vr ← res_V & val vr.1 \notin impacted_V' mod_V].
```

This allows us to form a final sequence of vertex-result pairs:

```
Def res_V' : seq (V' * R) := res_impacted_fresh_V' ++ res_unimpacted_V'.
```

For sanity-checking, we prove the absence of duplicates:

```
Def chk_V' : seq V' := [seq vr.1 | vr ← res_V'].
Thm chk_V'_uniq : uniq chk_V'.
```

We prove that the sequence contains all checkable vertices in `V'` (completeness):

```
Thm chk_V'_complete (v : V') : checkable' v → v \in chk_V'.
```

Finally, we prove that the results in the sequence are consistent with explicitly calling `check'` on all vertices in `V'` (soundness):

```
Thm chk_V'_sound (v : V') (r : R) : (v, r) \in res_V' → checkable' v ∧ check' v = r.
```

The formal proofs, which we elide here, mostly reduce to reasoning over the `connect` predicate and inductively on graph paths.

### 5 Component Hierarchies

Let $V$ be a set of vertices representing *fine-grained* components (e.g., methods), with dependency graph $g_\perp$. Let $U$ be a different set of vertices representing *coarse-grained* components (e.g., files), associated with a function $p\colon U \rightarrow 2^V$ that defines a *partition* of $V$. The partition indicates how components in $U$ *encapsulate* components in $V$, and is associated with a graph $g_\top$ of vertices in $U$ that is

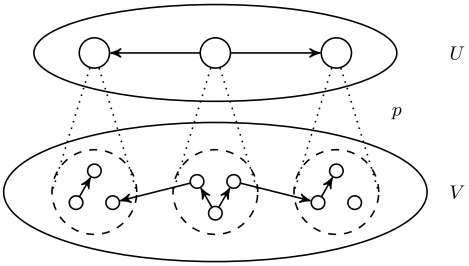

**Fig. 2.** Hierarchy with component sets $U$ and $V$, partition $p$, and dependencies.

consistent with dependencies expressed in $g_\bot$. This approach can be repeated to produce component *hierarchies*, each time coalescing sets of finer-grained dependencies into single coarser-grained dependencies. Figure 2 illustrates a two-level hierarchy and its component dependencies.

Some change impact analysis techniques consider both fine-grained and coarse-grained component levels [11, 46, 60]. A key idea behind these techniques is to exploit the relationships between vertices across granularity levels. In particular, if a vertex $u \in U$ is unmodified after a change, we may be able to immediately conclude that all vertices $v \in p(u)$ are unmodified as well, potentially ruling out that a large subset of $V$ is impacted. In this section, we formalize this intuition using our existing notions to express hierarchical change impact analysis.

#### 5.1 Formal Model of Hierarchies

Let $f_\bot$ and $f'_\bot$ be the functions mapping vertices to artifacts for $V$ and $V'$ with $V \subseteq V'$, and let $f_\top$ and $f'_\top$ be the corresponding functions for $U$ and $U'$ with $U \subseteq U'$. Let $p$ and $p'$ be partition-inducing functions from $U$ and $U'$ to subsets of $V$ and $V'$, respectively. We make the following assumptions:

H1: For all $u, u' \in U$ and $v, v' \in V$, if $u \neq u'$, $g_\bot(v, v')$, $v \in p(u)$, and $v' \in p(u')$, then $g_\top(u, u')$.

H2: For all $u \in U$, if $f_\top(u) = f'_\top(u)$, then $p(u) = p'(u)$.

H3: For all $u \in U$ and $v \in V$, if $f_\top(u) = f'_\top(u)$ and $v \in p(u)$, then $f_\bot(v) = f'_\bot(v)$.

Intuitively, H1 expresses that whenever two fine-grained components that reside in different coarse-grained components are related, there must be a corresponding relation between their respective coarse-grained components. H2 expresses that whenever a coarse-grained component is unchanged, it contains the same fine-grained components as before. Finally, H3 expresses that a fine-grained component is unchanged if the coarse-grained component that contains it is unchanged. Under these assumptions, there are essentially two distinct strategies we can use to leverage impact analysis for coarse-grained components to analyze fine-grained components.

**Overapproximation strategy**: Let $U'_i$ be the set of impacted and fresh vertices in $U'$, computed as above without considering vertices in $V'$. Consider the set $V'_p = \bigcup_{u \in U'_i} p'(u)$ which contains fresh and *potentially* impacted vertices in $V'$.

Executing all checkable vertices in $V_p'$ may perform needless work for unimpacted vertices, but completely elides analysis of $g_\bot$. This approach essentially corresponds to relying on comparing whole files to decide whether to rerun commands that operate on every component inside these files, as in `make`.

**Compositional strategy**: Let $U_i$ be the set of impacted vertices in $U$, computed as above. Consider the set $V_p = \bigcup_{u \in U_i} p(u)$ of potentially impacted vertices in $V$. We use this set to scope further analysis. In particular, we use the subgraph $g_p$ of $g_\bot$ induced by $V_p$ to precisely find the impacted vertices in $V$. While unimpacted vertices are then avoided, the additional analysis of $g_p$ may be time-consuming to perform compared to the first strategy. At a high level, this strategy corresponds to the one used in RPS [11] and hybrid RTS [60].

#### 5.2 Encoding and Correctness in Coq

To encode hierachical analysis, we use finite types and functions (now suffixed by `top` and `bot`) in the same way as before, while adding partitioning assumptions:

```
Vars (p : U → {set V}) (p' : U' → {set V'}).
Hyp p_pt : partition (\bigcup_(u | u \in U) [set (p u)]) [set: V].
Hyp p'_pt : partition (\bigcup_(u | u \in U') [set (p' u)]) [set: V'].
```

For the overapproximation strategy, we first define impacted sets:

```
Def if_top : {set U'} := impacted_fresh_V' f'_top f_top g_top.
Def p'_if_bot : {set V'} := \bigcup_( u | u \in if_top ) (p' u).
```

Under the assumptions outlined above, we then show formally that `p'_if_bot` is a superset of the results of analysis of `V`, `V'`, and the graph `g_bot`:

```
Thm in_p' (v : V') : v \in impacted_fresh_V' f'_bot f_bot g_bot → v \in p'_if_bot.
```

The key fact we use to prove this theorem is the following:

```
Thm connect_top_bot v v' u u' : v \in (p u) → v' \in (p u') →
 connect g_bot v v' → connect g_top u u'.
```

To encode the compositional strategy, we first define impacted sets:

```
Def i_top : {set U} := impacted g_top^-1 (mod_V f'_top f_top).
Def p_i_bot : {set V} := \bigcup_( u | u \in i_top ) (p u).
```

Then, we define a subtype and accompanying graph:

```
Def P_V_sub : pred V := fun v ⇒ v \in p_i_bot.
Def V_sub : finType := sig_finType P_V_sub.
Def g_bot_sub : rel V_sub := [rel x y | g_bot (val x) (val y)].
```

This allows us to use our previously defined analysis functions compositionally:

```
Def mod_V_sub := [set v : V_sub | val v \in mod_V f'_bot f_bot].
Def impacted_V_sub := impacted g_bot_sub^-1 mod_V_sub.
Def impacted_V'_sub := [set val (val v) | v in impacted_V_sub].
Def impacted_fresh_V'_sub := impacted_V'_sub :|: fresh_V' P_bot.
```

We finally show that the last set is the same as the one we would have obtained by directly analysing the graph `g_bot`:

```
Thm impacted_fresh_V'_sub_eq :
 impacted_fresh_V'_sub = impacted_fresh_V' f'_bot f_bot g_bot.
```

Using these definitions and results, we proved soundness and completeness for both strategies using the same approach as in Section 4.2.

### 6 Tool Implementation

While our core definitions of change impact analysis described in Section 4 are executable inside Coq, this does not mean they are efficient or that code extracted from the definitions is immediately usable. We describe two aspects of bringing verified Coq code into our tool CHIP: optimizations and encapsulation.

#### 6.1 Optimizations

Our basic transitive closure function `impacted` is simple to reason about but not particularly fast in practice, since it fully explores the closures of *all* elements in the set of modified vertices. To mitigate this, we refined the function by leveraging the depth-first search function `dfs` from the `fingraph` MC module to incrementally compute the closure. `dfs` takes a graph as a function from vertices to neighbor sequences and a depth bound, and terminates as soon as it encounters a known vertex. We perform a stack-efficient left fold with `dfs` over an input sequence of vertices:

```
Def clos (l : seq V) : seq V := foldl (dfs g #|V|) [::] l.
```

Note that we set the `dfs` depth bound to the number of elements in the finite type `V` (written #|`V`|) to fully explore the graph `g`. However, one limitation of the MC `dfs` function is its linear-time sequence membership lookups. We therefore defined a better closure function with logarithmic membership lookup time using sets backed by red-black trees as found in the Coq standard library [2, 23]:

```
Fixpoint sdfs (g : V → seq V) (n : nat) (s : RBT.t) (x : V) : RBT.t :=
 if RBT.mem x s then s else
 if n is n'.+1 then foldl (sdfs g n') (RBT.add x s) (g x) else s.
Def sclos (l : seq V) : seq V := RBT.elements (foldl (sdfs g #|V|) RBT.empty l).
```

We used this closure function to define a function `seq_impacted_fresh` which we proved extensionally equivalent to `impacted_fresh_V'` defined in Section 4.1. We also added many custom extraction directives in Coq to ensure the extracted code uses efficient OCaml library functions, e.g., for list operations [22].

#### 6.2 Encapsulation

Before extraction to OCaml, we instantiate the finite types for graph vertices to *ordinal* finite types, which intuitively contain all natural numbers from 0 up to (but not including) some bound $k$. These numbers can then become machine integers during extraction, which allows us to provide a simple OCaml interface:

```
val impacted_fresh : int -> int -> (int -> string) ->
  (int -> string) -> (int -> int list) -> int list
```

Here, the first argument is the number of vertices in the new graph, while the second is the number of vertices in the old graph. After these integers follow two functions that map new and old vertices, respectively, to their artifacts in the form of OCaml strings. Then comes a function that defines the adjacent vertices of vertices in the old graph. The result is a list of impacted and fresh vertices.

Not all computationally meaningful types in Coq can be directly represented in OCaml's type system. Some function calls must therefore *circumvent* the type system by using calls to the special `Obj.magic` function [40]. We use this approach in our implementation of the above interface:

```
let impacted_fresh num_new num_old f' f succs =
 Obj.magic (ordinal_seq_impacted_fresh num_new num_old
 (Obj.magic (fun x -> char_list_of_string (f' x)))
 (Obj.magic (fun x -> char_list_of_string (f x)))
 (Obj.magic succs))
```

The interface and implementation for two-level compositional hierarchical selection is a straightforward extension, with an additional argument `p` of type `int -> int list` for between-level partitioning.

## 7 Evaluation of the Model

To evaluate our model and its Coq encoding, we performed an empirical study by integrating Chip with a recently developed RTS tool, Ekstazi, one RPS tool, iCoq, and one build system, Tup. We then ran the modified RTS tool on open-source Java projects used to evaluate RTS techniques [26,37], the modified RPS tool on Coq projects used in its evaluation [11], and the modified build system on C/C++ projects. Finally, we compared the outcomes and running times with those for the unmodified versions of Ekstazi, iCoq, and Tup.

### 7.1 Tool Integration

Integrating Chip with Ekstazi was challenging, since Ekstazi collects dependencies dynamically and builds only a flat list of dependencies rather than an explicit graph. To overcome this limitation, we modified Ekstazi to build an explicit graph by maintaining a mapping from method callers to their callees. The integration with iCoq was also challenging because of the need for hierarchical selection of proofs and support for deletion of dependency graph vertices. We handle deletion of a vertex in iCoq by temporarily adding it to the new graph with a different artifact (checksum) from before, marked as non-checkable; then, after selection, we purge the vertex. In contrast, the integration with Tup was straightforward, since Tup stores dependencies in an SQLite database. We simply query this database to obtain a graph in the format expected by Chip.

### 7.2 Projects

**RTS**: We use 10 GitHub projects. Table 1 (top) shows the name of each project, the number of lines of code (LOC) and the number of tests in the latest version control revision we used in our experiments, the SHA of the latest revision, and

**Table 1.** List of Projects Used in the Evaluation (RTS at the Top, RPS in the Middle, and Tup at the Bottom).

| **Project** | **LOC** | **#Tests** | **SHA** | **URL (github.com/)** |
|---|---|---|---|---|
| Asterisk | 57,219 | 257 | e36c655f | asterisk-java/asterisk-java |
| Codec | 22,009 | 887 | 58860269 | apache/commons-codec |
| Collections | 66,356 | 24,589 | d83572be | apache/commons-collections |
| Lang | 81,533 | 4,119 | c3de2d69 | apache/commons-lang |
| Math | 186,388 | 4,858 | eb57d6d4 | apache/commons-math |
| GraphHopper | 70,615 | 1,544 | 14d2d670 | graphhopper/graphhopper |
| La4j | 13,823 | 799 | e77dca70 | vkostyukov/la4j |
| Planner | 82,633 | 398 | f12e8600 | opentripplanner/OpenTripPlanner |
| Retrofit | 20,476 | 603 | 7a0251cc | square/retrofit |
| Truth | 29,591 | 1,448 | 14f72f73 | google/truth |
| Total | 630,643 | 39,502 | N/A | N/A |
| **Project** | **LOC** | **#Proofs** | **SHA** | **URL** |
| Flocq | 33,544 | 943 | 4161c990 | gitlab.inria.fr/flocq/flocq |
| StructTact | 2,497 | 187 | 8f1bc10a | github.com/uwplse/StructTact |
| UniMath | 45,638 | 755 | 5e525f08 | github.com/UniMath/UniMath |
| Verdi | 57,181 | 2,784 | 15be6f61 | github.com/uwplse/Verdi |
| Total | 138,860 | 4,669 | N/A | N/A |
| **Project** | **LOC** | **#Cmds** | **SHA** | **URL (github.com/)** |
| guardcheader | 656 | 5 | dbd1c0f | kalrish/guardcheader |
| LazyIterator | 1,276 | 18 | d5f0b64 | matthiasvegh/LazyIterator |
| libhash | 347 | 10 | b22c27e | fourier/libhash |
| Redis | 162,366 | 213 | 39c70e7 | antirez/redis |
| Shaman | 925 | 7 | 73c048d | HalosGhost/shaman |
| Tup | 200,135 | 86 | f77dbd4 | gittup/tup |
| Total | 365,705 | 339 | N/A | N/A |

URL on GitHub. We chose these projects because they are popular Java projects (in terms of stars) on GitHub, use the Maven build system (supported by Ekstazi), and were recently used in RTS research [37, 60].

**RPS**: We use 4 Coq projects. Table 1 (middle) shows the name of each project, the number of LOC and the number of proofs in the latest revision we used, the latest revision SHA, and URL. We chose these projects because they were used in the evaluation of iCoq [11]; as in that evaluation, we used 10 revisions of StructTact and 24 revisions of the other projects.

**Build system**: We use 6 GitHub projects. Table 1 (bottom) shows the name of each project, the number of LOC and the number of build commands in the latest revision we used, the latest revision SHA, and URL. We chose these projects from the limited set of projects on GitHub that use Tup. We looked for projects that could be built successfully and had at least five revisions; the largest project that met these requirements, in terms of LOC, was Tup itself.

#### 7.3 Experimental Setup

Our experimental setup closely follows recent work on RTS [37, 60]. That is, our scripts (1) clone one of the projects; (2) integrate the (modified) Ekstazi, iCoq, or Tup; and (3) execute tests on, check proofs for, or build the (up to) 24 latest revisions. For each run, we recorded the end-to-end execution time, which includes time for the entire build run. We also recorded the execution time for change impact analysis alone. Finally, we recorded the number of executed tests,

**Table 2.** Execution Time and CIA Time in Seconds for Ekstazi and Chip.

| Project | Total | | | CIA | |
|---|---|---|---|---|---|
| | RetestAll | Ekstazi | Chip | Ekstazi | Chip |
| Asterisk | 461.92 | 188.67 | 194.65 | 2.74 | 6.51 |
| Codec | 896.00 | 135.11 | 136.35 | 2.44 | 4.10 |
| Collections | 2,754.99 | 342.07 | 350.95 | 2.87 | 9.31 |
| Lang | 1,844.19 | 359.36 | 367.16 | 2.71 | 8.68 |
| Math | 2,578.09 | 1,459.98 | 1,495.71 | 1.79 | 7.13 |
| GraphHopper | 1,871.01 | 423.63 | 449.94 | 11.19 | 21.33 |
| La4j | 272.96 | 202.10 | 209.41 | 1.12 | 3.91 |
| Planner | 4,811.63 | 1,144.09 | 1,228.61 | 40.62 | 89.17 |
| Retrofit | 1,181.09 | 722.14 | 747.76 | 11.30 | 19.97 |
| Truth | 745.11 | 700.26 | 724.22 | 3.03 | 8.82 |
| Total | 17,416.99 | 5,677.41 | 5,904.76 | 79.81 | 178.93 |

**Table 3.** Execution/CIA Time in Seconds for iCoq and Chip.

| Project | Total | | | CIA | |
|---|---|---|---|---|---|
| | RecheckAll | iCoq | Chip | iCoq | Chip |
| Flocq | 1,028.01 | 313.08 | 318.19 | 50.65 | 53.43 |
| StructTact | 45.86 | 43.90 | 44.49 | 14.45 | 14.98 |
| UniMath | 14,989.09 | 1,910.56 | 2,026.75 | 124.79 | 239.12 |
| Verdi | 37,792.07 | 3,604.23 | 4,637.27 | 139.09 | 1,171.57 |
| Total | 53,855.03 | 5,871.76 | 7,026.70 | 328.98 | 1,479.10 |

proofs, or commands, which we use to verify the correctness of the results, i.e., we checked that the results for the unmodified tool and Chip were equivalent. We ran all experiments on a 4-core Intel i7-6700 CPU @ 3.40GHz machine with 16GB of RAM, running Ubuntu Linux 17.04. We confirmed that the execution time for each experiment was similar across several runs.

#### 7.4 Results

**RTS**: Table 2 shows the execution times for Ekstazi. Column 1 shows the names of the projects. Columns 2 to 4 show the cumulative end-to-end time for RetestAll (i.e., running all tests at each revision), the unmodified RTS tool, and the RTS tool with Chip. Columns 5 and 6 show the cumulative time for change impact analysis (CIA time). The last row in the table shows the cumulative execution time across all projects. We have several findings. First, Ekstazi with Chip performs significantly better than RetestAll, and only slightly worse than the unmodified tool. Considering that we did not prioritize optimizing the integration, we believe that the current execution time differences are small. Second, the CIA time using Chip is slightly higher than the CIA time for the unmodified tool, but we believe this could be addressed by integrating Chip via the Java Native Interface (JNI). The selected tests for all projects and revisions were the same for the unmodified Ekstazi and Ekstazi with Chip.

**RPS**: Table 3 shows the total proof checking time for iCoq and the CIA time for iCoq and Chip. All time values are cumulative time across all the revisions we used. We find that iCoq with Chip has only marginal differences in performance from iCoq for all but the largest project, Verdi. While iCoq with

Chip is notably slower in that case, it still saves a significant fraction of time from checking every revision from scratch (RecheckAll). StructTact is an outlier in that RecheckAll is actually faster than both iCoq and iCoq with Chip, due to the overhead from bookkeeping and graph processing in comparison to the project's relatively small size. The selected proofs for all projects and revisions were the same for the unmodified iCoq and iCoq with Chip.

**Build system**: Table 4 shows the total execution time for Tup and the CIA time for Tup and Chip. All time values are cumulative time across all the revisions we used. Unfortunately, the build time for most of the projects is short. However, we can still observe that Chip takes only slightly more time than the original tool to perform change impact analysis. In the future, we plan to evaluate our toolchain on larger projects. The lists of commands for all projects and all revisions were the same for the unmodified Tup and Tup with Chip.

**Table 4.** Execution Time in Milliseconds for Tup and Chip.

| Project | Tup | CIA Tup | CIA Chip |
|---|---|---|---|
| guardcheader | 20,358 | 1,788 | 1,785 |
| LazyIterator | 61,476 | 869 | 1,007 |
| libhash | 15,279 | 433 | 446 |
| Redis | 68,076 | 1,919 | 4,779 |
| Shaman | 8,702 | 609 | 614 |
| Tup | 87,547 | 1,949 | 4,168 |
| Total | 261,438 | 7,567 | 12,799 |

Overall, we believe these results indicate that our formal model is practically relevant and that it is feasible to use Chip as a verified component for change impact analysis in real-world tools.

### 8 Related Work

**Formalizations of graph algorithms**: Pottier [49] encoded and verified Kosaraju's algorithm for computing strongly connected graph components in Coq. He also derived a practical program for depth-first search by extracting Coq code to OCaml, demonstrating the feasibility of extraction for graph-based programs. Théry subsequently formalized a similar encoding of Kosaraju's algorithm in Coq using the MC `fingraph` module [56]. Théry and Cohen then formalized and proved correct Tarjan's algorithm for computing strongly connected graph components in Coq [13,16]. Our formalization takes inspiration from Théry and Cohen's work, and adapts some of their definitions and results in a more applied context, with focus on performance of extracted code. Similar graph algorithm formalizations have also been done in the Isabelle/HOL proof assistant [35]. In work particularly relevant to build systems, Guéneau et al. [32] verified both the functional correctness and time complexity of an incremental graph cycle detection algorithm in Coq. In contrast to our reasoning on pure functions and use of extraction, they reason directly on imperative OCaml code.

**Formalizations of build systems**: Christakis et al. [15] formalized a general build language called CloudMake in the Dafny verification tool. Their language is a purely functional subset of JavaScript, and allows describing dependencies between executions of tools and files. Having embedded their language in Dafny, they verify that builds with cached files are equivalent to builds from scratch. In contrast to the focus on generating files in CloudMake, we consider a formal model with an explicit dependency graph and an operation *check* on vertices whose output is not used as input to other operations. The CloudMake formalization assumes an arbitrary operation *exec* that can be instantiated using

Dafny's module refinement system; we use Coq section variables to achieve similar parametrization for *check*. We view our Coq development as a *library* useful to tool builders, rather than a separate language that imposes a specific idiom for expressing dependencies and build operations.

Mokhov et al. [45] presented an analysis of several build systems, including a definition what it means for such systems to be correct. Their correctness formulation is similar to that of Christakis et al. for cached builds, and relies on a notion of abstract persistent stores expressed via monads. Our vertices and artifacts correspond quite closely to their notions of *keys* and *values*, respectively. However, their basic concepts are given as Haskell code, which has less clear meaning and a larger trusted base than Coq or Dafny code. Moreover, they provide no formal proofs. Mokhov et al. [44] subsequently formalized in Haskell a static analysis of build dependencies as used in the Dune build system.

Stores could be added to our model, e.g., by letting checkable vertices be associated with commands that take lists of file names and the current store state as parameters, producing a new state. However, this would in effect entail defining a specific build language inside Coq, which we consider outside the scope of our library and tool.

### 9 Conclusion

We presented a formalization of change impact analysis and its encoding and correctness proofs in the Coq proof assistant. Our formal model uses finite sets and graphs to capture system components and their interdependencies before and after a change to a system. We locate impacted vertices that represent, e.g., tests to be run or build commands to be executed, by computing transitive closures in the pre-change dependency graph. We also considered two strategies for change impact analysis of hierarchical systems of components. We extracted optimized impact analysis functions in Coq to executable OCaml code, yielding a verified tool dubbed Chip. We then integrated Chip with a regression test selection tool for Java, Ekstazi, one regression proof selection tool for Coq itself, iCoq, and one build system, Tup, by replacing their existing components for impact analysis. We evaluated the resulting toolchains on several open-source projects by comparing the outcome and running time to those for the respective original tools. Our results show the same outcomes with only small differences in running time, corroborating the adequacy of our model and the feasibility of practical verified tools for impact analysis. We also believe our Coq library can be used as a basis for proving correct domain-specific incremental techniques that rely on change impact analysis, e.g., regression test selection for Java and regression proof selection for type theories.

### Acknowledgments

The authors thank Ben Buhse, Cyril Cohen, Pengyu Nie, Zachary Tatlock, Thomas Wei, Chenguang Zhu, and the anonymous reviewers for their comments and feedback on this work. This work was partially supported by the US National Science Foundation under Grant No. CCF-1652517.
